# Genome-wide CRISPR screen identifies neddylation as a regulator of neuronal aging and AD neurodegeneration

**DOI:** 10.1016/j.stem.2025.12.019

**Published:** 2026-01-03

**Authors:** Nathalie Saurat, Andrew P. Minotti, Maliha T. Rahman, Trisha Sikder, Chao Zhang, Daniela Cornacchia, Johannes Jungverdorben, Gabriele Ciceri, Doron Betel, Lorenz Studer

Our original manuscript erroneously listed the concentration of SB431542 as 10 mM instead of 10 μM in the dopamine neuron differentiation section of the Method details section. We apologize for this error, which has been corrected in the original version.

The corrected manuscript also includes additional changes to comply with the open access policy requirements of the funder (Aligning Science Across Parkinson’s disease). These include more detailed information listed in the key resource table, links to detailed protocols and code used in the study, and access to source data for all figures and supplemental information (which was added as Data S1 in the supplemental files). None of the changes made in this correction affect the conclusions of the manuscript.

## Supplementary Material

Data S1 (Raw Data)

Data S1 (Readme file)

## Figures and Tables

**Table:** 

REAGENT or RESOURCE	SOURCE	IDENTIFIER
Antibodies		

Mouse anti β-ACTIN	Sigma	Cat# A2228–100UL RRID: AB_476697
Mouse anti BAG1	Santa Cruz Biotech	Cat# sc-33704 RRID: AB_626720
Rabbit anti BAG3	Abcam	Cat# ab47124, RRID: AB_867788
Mouse anti Cas9	Cell Signaling Tech	Cat# 14697S, RRID: AB_2750916
Mouse anti Ki67	Dako	Cat# M7240, RRID: AB_2142367
Mouse anti GAPDH-HRP	Santa Cruz Biotech	Cat# sc-47724 RRID: AB_627678
Rabbit anti LMNB1	Abcam	Cat# ab16048, RRID: AB_443298
Rabbit anti NAE1	Cell Signaling Tech	Cat# 14321, RRID: AB_2798448
Rabbit anti NEDD8	Abcam	Cat# ab81264, RRID: AB_1640720
Rabbit anti p21	Cell Signaling Tech	Cat# 2947, RRID: AB_823586
Mouse anti TAU	Thermo Fisher	Cat# MN1000 RRID: AB_2314654
Mouse anti p-TAU(S202/T205)	Thermo Fisher	Cat# MN1020, RRID: AB_223647
Rabbit anti UBA3	Abcam	Cat# ab124728, RRID: AB_10974248
Mouse anti phospho-ATM(S1981)	Thermo Fisher	Cat# MA1–2020, RRID: AB_1086244
Rabbit anti Cleaved Caspase-3 (Asp175)	Cell Signaling Tech	Cat# 9661, RRID: AB_2341188)
Mouse anti p53	Cell Signaling Tech	(Cat# 48818, RRID: AB_2713958)
Rabbit anti FOXG1	TaKaRa	Cat# M227 RRID: AB_2827749
Chicken anti MAP2	Thermo Fisher	Cat# PA1–16751 RRID: AB_2138189
Rabbit anti NANOG	Thermo Fisher	Cat# 4903, RRID: AB_10559205
Rabbit anti PAX6	Biolegend	Cat# 901301 RRID: AB_2749901
Mouse anti SENP8	Santa Cruz Biotech	Cat# sc-271498 RRID: AB_10649498
Mouse anti p62 Lck Ligand	BD Bioscience	Cat# 610832 RRID: AB_398151
Rabbit anti LC3A/B XP	Cell Signaling Tech	Cat# 12741S RRID: AB_2617131
Rabbit p-TAU(S235)	Thermo Fisher	Cat# PA5–104785, RRID: AB_2816258
Mouse anti TAU	Dako	Cat# A0024, RRID: AB_10013724
Mouse anti Caveolin 1 (CAV1)	Thermo Fisher	Cat# MA3–600, RRID: AB_779568
Rabbit anti H3K9me3	Abcam	Cat# ab176916 RRID: AB_2797591
Mouse anti Lap2	BD Bioscience	Cat# 611000 RRID: AB_398313
Rabbit anti H3k9me3-PE	Cell Signaling Tech	Cat# 13969S RRID: AB_2798355
Anti-mouse IgG, HRP-linked	Cell Signaling Tech	Cat#7076S RRID: AB_330924
Anti-rabbit IgG, HRP-linked	Cell Signaling Tech	Cat#7074S, RRID: AB_2099233
Donkey anti-mouse IgG H&L (Alexa Fluor^®^488)	Invitrogen	Cat# R37114, RRID: AB_2556542
Donkey anti-rabbit IgG H&L (Alexa Fluor^®^488)	Invitrogen	Cat# A21206, RRID: AB_2535792
Donkey anti-mouse IgG H&L (Alexa Fluor^®^555)	Invitrogen	Cat# A31570, RRID: AB_2536180
Donkey anti-rabbit IgG H&L (Alexa Fluor^®^555)	Invitrogen	Cat# A31572 RRID: AB_162543
Donkey anti-chicken IgG H&L (Alexa Fluor^®^647)	Jackson ImmunoResearch	Cat# 703–605-155 RRID: AB_2340379
Goat anti-mouse IgG H&L (Alexa Fluor^®^488)	Invitrogen	Cat# A11029 RRID: AB_2534088
Goat anti-rabbit IgG H&L (Alexa Fluor^®^488)	Invitrogen	Cat# A11034 RRID: AB_2576217
Goat anti-mouse IgG H&L (Alexa Fluor^®^555)	Invitrogen	Cat# A21422 RRID: AB_141822
Goat anti-rabbit IgG H&L (Alexa Fluor^®^555)	Invitrogen	Cat# A27039 RRID: AB_2536100
Goat anti-chicken IgG H&L (Alexa Fluor^®^647)	Invitrogen	Cat# A21449 RRID: AB_2535866

Bacterial and virus strains		

One Shot^™^ Stbl3^™^ Chemically Competent E. coli	Thermo Fisher	C737303

Chemicals, peptides, and recombinant proteins		
Halt Protease Inhibitor	Thermo Fisher	87786
Halt Phosphatase inhibitor	Thermo Fisher	78420
Y-27632	R&D Systems	1254
SB431542	R&D Systems	1614
LDN193189 (LDN)	Stemgent	04–0074
CHIR99021	R&D Systems	4432
SHH C25II	R&D Systems	464-SH
Poly-L-Ornithine (PO)	Sigma Aldrich	P3655
Mouse Laminin I (LAM)	R&D Systems	3400–010-1
Fibronectin (FN)	Thermo Fisher Scientific	356008
0.5M EDTA, pH 8.0	Thermo Fisher Scientific	15575020
Accutase	Innovative Cell Technologies	AT104–500
Trypsin-EDTA 0.05%	Thermo Fisher Scientific	25300–054
GDNF	Peprotech	450–10
AA	Sigma	4034–100g
cAMP	Sigma	D0627
BDNF	R&D Systems	248-BD
TGFβ3	R&D Systems	243-B3
DAPT	R&D Systems	2634
DMEM:F12 w/high glucose	Thermo Fisher Scientific	11320–033
E8 medium	Thermo Fisher	A1517001
E6 medium	Thermo Fisher	A1516401
DMEM/F12	Thermo Fisher	21331020
Neurobasal medium	Life Technologies	21103–049
100X N2 supplement	Life Technologies	17502–048
50X B27 supplement w/o Vit A	Life Technologies	12587–010
L-Glutamine (100X)	Thermo Fisher Scientific	25030–081
Penicillin Streptomycin	Thermo Fisher Scientific	15140–122
4’,6-diamidino-2-phenylindole (DAPI)	Sigma	D9542
Matrigel	BD Biosciences	356234
Vitronectin (VTN-N)	Thermo Fisher Scientific	A14700
Geltrex	Life Technologies	A1413201
Trizol	Thermo Fisher Scientific	15596026
STEM-CELLBANKER	Amsbio	11890
4% paraformaldehyde	Affymetrix	MFCD00133991
Neuron Isolation Enzyme	Thermo Fisher Scientific	88285
Precision Red Advanced Protein Assay	Cytoskeleton, Inc	ADV02-A
DPBS	Thermo Fisher Scientific	14190–136
CellEvent Senescence Green	Thermo Fisher Scientific	C10840
X-tremeGENE HP	Sigma	6366236001
Doxycycline hyclate	Sigma	D9891
Proteostat	Enzo Life Sciences	ENZ-51023-KP050
MLN4924 (Pevonedistat)	MedChemExpress	HY-70062
CSN5i3	Tocris	7089
AggreSure ß-Amyloid (1–42), human	AnaSpec	AS-72216
MG132	Selleck	S2619
Bafilomycin A1	Cayman Chemical	11038
N-Lauroylsarcosine sodium salt solution	Sigma	L7414–10ML

Critical commercial assays		

Direct-zol RNA Microprep	Zymo Research	R2062
QiAquick gel extraction kit 250	Qiagen	28706
QIAprep Spin Miniprep Kit	Qiagen	27106
NucleoBond Xtra Midi Plus EF	Macherey-Nagel	740422.50
iScript^™^ Reverse Transcription Supermix for RT-qPCR	Biorad	1708841
SsoFast^™^ EvaGreen^®^ Supermix	Biorad	1725201
NuPAGE^™^ 4–12% Bis-Tris Protein Gels, 1.5 mm, 15-wells	Thermo Scientific	NP0336BOX
NuPAGE^®^ Novex 4–12% Bis-Tris Gel 1.5 mm, 10 well	Thermo Scientific	NP0335Box
NuPAGE^®^ Novex^®^ 4–12% Bis-Tris Gels, 1.0 mm, 12 wel	Thermo Scientific	NP0322BOX
Zombie UV^™^ Fixable Viability Kit	Biolegend	423107
Cell Counting Kit - 8	Sigma Aldrich	96992–100TESTS-F
PrestoBlue Cell Viability Reagent	Thermo	A13261
V-PLEX Aβ Peptide Panel 1 (6E10) Kit	Meso Scale Discovery	K15200E-2
Pierce Reversible Protein Stain Kit for PVDF Membranes	Thermo Fisher Scientific	24585
Cell-Based Proteasome-Glo^™^ Assay kit	Promega	G8660

Deposited data		

RNAseq data. Young and Old Cortex	Zhang et al.^35^	PRJNA1107241
Tabula Muris Senis trajectory analysis	Schaum et al.^66^	GEO: GSE132040) https://twc-stanford.shinyapps.io/maca/
Young and Old Protein expression	Keele et al.^67^	Table S3; DOI: https://doi.org/10.1016/j.celrep.2023.112715
Essential genes in iNeurons	Tian et al.^19^	Table S1; DOI: https://doi.org/10.1038/s41593-021-00862-0
Essential genes in striatal neurons	Wertz et al.^18^	Table S2; DOI: https://doi.org/10.1016/j.neuron.2020.01.004
Essential gene catalogue	Project Achilles	CRISPRGeneEffect.csv; https://depmap.org/portal/download/all/

Experimental models: Cell lines		

H9 derived iCas9 cell line	This study	RRID: CVCL_E7G4
H9 derived iCas9 cell line with APPswe/swe mutation	This study	RRID: CVCL_E7G5
7889SA iCas9 cell line	This study	RRID: CVCL_E7GB
7889SA iCas9 cell line with PSEN^M146V/M146V^ mutation	This study	RRID: CVCL_E7GC
H9 derived Nurr1::H2B-GFP iCas9	Kim et al.^68^	RRID: CVCL_E7GJ
H9 derived Nurr1::H2B-GFP iCas9 with the LRRK2^G2019S/G2019S^ mutation	This study	RRID: CVCL_E7GK

Oligonucleotides		

qPCR Primers: See Table S2	This study	N/A
Cloning Primers: See Table S2	This study	N/A
Oligonucleotides for Cloning: See Table S2	This study	N/A
sgRNAs: See Table S3	Doench et al^69^	Table S3; DOI: https://doi.org/10.1038/nbt.3437

Recombinant DNA		

Hygro-Cas9 donor	Shi et al., 2017^70^	RRID: Addgene_86883
Neo-M2rtTA donor	Gonzalez et al.^12^	Danwei Huangfu Lab; MSKCC
AAVS1-TALEN-L	Gonzalez et al.^12^	RRID: Addgene_59025
AAVS1-TALEN-R	Gonzalez et al.^12^	RRID: Addgene_59026
lentiGuide-Puro	Sanjana et al.^71^	RRID: Addgene_52963
Brunello sgRNA library	Doench et al.^69^	RRID: Addgene_73178
psPAX2	Didier Trono lab	RRID: Addgene_12260
pMD2.G	Didier Trono lab	RRID: Addgene_12259
pLV-EF1a-IRES-Puro	Hayer et al.^72^	RRID: Addgene_85132
UBA3 ORF	Sino Biological	Cat# HG16320-G
NAE1 ORF	Sino Biological	Cat # HG14282-G
pLV-EF1aNAE1-IRES-Puro	This study	RRID: Addgene_#235659
pLV-EF1aUBA3-IRES-Puro	This study	RRID: Addgene_#235660

Software and algorithms		

FIJI	Schindelin et al.^73^	https://fiji.sc/ RRID: SCR_003070
Volocity Imaging Software	Perkin Elmer	http://www.perkinelmer.com/pages/020/cellularimaging/products/volocity.xhtml RRID: SCR_002668
FlowJo	FlowJo	https://www.flowjo.com/ RRID: SCR_008520
R		https://cran.r-project.org/ RRID: SCR_001905
Prism 9	GraphPad	www.graphpad.com RRID: SCR_002798
Biorender	Biorender	http://biorender.com RRID: SCR_018361
MAGECK-MLE	Wang et al.^17^	https://mageck.sourceforge.net/ RRID: SCR_025016
Python		https://www.python.org RRID: SCR_008394
Image Lab Software	Bio-Rad	http://www.bio-rad.com/en-us/sku/1709690-image-lab-software RRID: SCR_014210
DAVID (v6.8)	Huang da et al.^74^	https://david.ncifcrf.gov/summary.jsp RRID: SCR_001881
Harmony	Revvity	https://www.revvity.com/product/harmony-5-2-office-revvity-hh17000019 RRID: SCR_023543
ggplot2		https://cran.r-project.org/web/packages/ggplot2/index.html RRID: SCR_014601
GSEA 3.0		http://software.broadinstitute.org/gsea/index.jsp RRID: SCR_003199

