# Supplementary material for: Genome-wide CRISPR screen identifies neddylation as a regulator of neuronal aging and AD neurodegeneration
Source: Cell Stem Cell. Author manuscript; Available in PMC 2026 Mar 10. (PMC12973372; doi:10.1016/j.stem.2025.12.019)
Supplement: Data S1 (Readme file) [file NIHMS2134384-supplement-1.rtf]

README for resource file Saurat et al. 2024 Cell Stem Cell		https://doi.org/10.1016/j.stem.2024.06.001Fig. 1CqPRC quantification of Cas9 induction after 48 h treatment with doxycycline (n = 5). Control ESCs vs APPswe, control neurons at DIV22 vs APPswe neurons DIV22.Fig.1EQuantification of neurons and cycling contaminants at DIV 30 in % (n = 3). MAP2 for neurons, Ki67 cycling cells.Fig. 1FELISA quantification of amyloid peptide production at DIV 35 in pg/ml (n = 3, unpaired two-tailed t test). Total a-beta and a-beta 40:42.Fig. 1HDensitometric quantification of western blots for phospho-TauS202/T205 (AT8) and total Tau (T-Tau) in DIV 65 neurons. (n = 3). Fig. 2K+LPresto blue viability assay in (K) UBA3 or NAE1 knockout or MLN4924-treated (L) neurons ± addition of Aβ2. (n = 4, one-way ANOVA).Fig. 2M-OImmunofluorescence images and quantification of cleaved caspase 3 (Cl-cas3) and p53 after 10, 20, or 30 days of treatment with MLN4924 (one sample t test; n = 12, median and quartile range). One outlier was excluded from p53 analysis. Scale bars, 100 μ. Figure data represented as mean  SD unless otherwise stated.Fig. 3C-ERepresentative images and quantification of nuclear area and roundness of nuclei from MLN4924-treated neurons. Scale bars, 25 μ. n = 12.Fig. 3IWestern blotting and densitometric quantification of BAG1 and BAG3 with in MLN4924-treated neurons (paired t test; WT n = 3; APPswe n = 4).Fig. 3J-KProteasome activity after 10 or 20 days of treatment with MLN4924 (one sample t test; n = 3 or 4). WT and APPswe neurons.Fig. 3M-PRepresentative immunofluorescent images and quantification of H3K9me3, LAP2 and pATM from control vs. MLN4924-treated WT and APPswe neurons. Scale bars, 25 μ. One sample t test; n = 12.Fig. 4BQuantification of pTau(S235) or pTau(235)bright normalized to total Tau (mean ± SD, n = 3; unpaired two-tailed t test). DIV50.Fig. 4CFractionation and densitometric quantification of soluble and insoluble Tau by western blotting (median, n = 4; one-way ANOVA). DIV50
